# Academic, Leadership, and Demographic Characteristics of Orthopaedic Sports Medicine Division Chiefs in the United States

**DOI:** 10.5435/JAAOSGlobal-D-21-00139

**Published:** 2022-01-10

**Authors:** Noorullah Maqsoodi, Addisu Mesfin, Xinning Li

**Affiliations:** From the Department of Orthopaedic Surgery, Boston University School of Medicine, One Boston Medical Center Pl, Boston, MA (Dr. Maqsoodi and Dr. Li), and the Department of Orthopaedic Surgery, University of Rochester Medical Center, Rochester, NY (Dr. Mesfin).

## Abstract

**Introduction:**

Division chiefs (DCs) play an integral role within the department, making critical decisions and helping shape the future of both the division and the department. This study aimed to investigate the demographic characteristics and scholarly work of DCs in academic orthopaedic sports medicine division in the United States.

**Methods:**

Orthopaedic residency programs at academic centers were identified using the Association of American Medical Colleges' Electronic Residency Application Service. DCs were identified using the program's respective websites where data points such as sex, race/ethnicity, fellowship training institution, time since graduating fellowship, academic rank, number of degrees, and additional leadership titles were collected. Scopus database was used to determine h-indices.

**Results:**

From the 191 programs identified, 100 had a DC for the sports medicine subspecialty division, and 66 programs offered a sports medicine fellowship. Most DCs (96%) were men. The racial/ethnic demographics of the DCs were mostly White (86%), followed by Asian (11%), African American (1%), Hispanic/Latino (1%), and mixed ethnicity (1%). On average, the DCs were 19.6 years past their fellowship completion. The average h-index was 21.2. Many (48%) had an academic rank of professor, 28% associate professor, and 12% assistant professor. Four held additional graduate degrees. The fellowship programs that trained the most DCs were Hospital for Special Surgery (11), Kerlan Jobe Orthopaedic Clinic (8), University of Pittsburgh (7), American Sports Medicine Institution (5), Cleveland Clinic (5), Cincinnati Sports Medicine (4), Massachusetts General Hospital (4), and Steadman Hawkins Clinic (4).

**Discussion:**

DCs in academic orthopaedic surgery plays a crucial role in the department and is a topic that is understudied. A lack of diversity exists among DCs in academic Sports Medicine in orthopaedics. The position is held predominately by White men with a rank of either full or associate professor and extensive leadership experience. More efforts are needed to increase the diversity of sports medicine leadership within academic orthopaedic programs in the United States.

Sports medicine is an integral part of any orthopaedic surgery department. Orthopaedic sports medicine deals with examination, preservation, and restoration of the musculoskeletal system affected by athletic activity of all levels. They also provide care to athletes, sports teams, or active individuals that include both nonsurgical treatment and sideline coverage of sport competitions. The care for athletes is led through a multidisciplinary care team involving orthopaedic surgeons, primary care physicians, athletic trainers, physical therapists, conditioning experts, and others.^1^

A paucity of available publications that provide information about academic orthopaedic division chief (DC) leadership in the United States exists. A study of DC leadership is imperative because they often transition to vice-chairperson or chairperson of orthopaedic departments as the next step in their career. Other specialties including plastic surgery, infectious disease, cardiology, and pulmonary/critical care have evaluated the characteristics and demographics of the respective DCs.^2-4^

This study had two objectives: (1) to identify the academic characteristics of orthopaedic sports surgery DCs and (2) to evaluate the demographic characteristics of sports medicine DCs.

## Methods

The FRIEDA Residency Program Database was used to identify allopathic (MD) orthopaedics programs associated with an academic center. In April 2020, publicly available program websites were used to locate DC. From individual DC academic profile page, sex, academic rank, additional leadership position(s), additional degree(s), fellowship institution, and years since completion of fellowship were determined. The authors identified and selected the race through a web-based profile search and the ethnicity based on the last name. Furthermore, the profile picture of each DC was used to help confirm their ethnicity. The institutions' department page was used to determine the number of faculty in the department.

Traveling fellowships were recorded for each DC using each programs’ website. We queried fellowships offered by the American Orthopaedic Society for Sports Medicine (AOSSM). These included exchange programs to Europe, Latin American, and Asia. In addition, we included American Orthopaedic Association (AOA) traveling fellowships.

The Hirsch index (h-index), defined as a researchers' number of publications cited ≥h times, is used to measure scholarly productivity.^5^ For comparison purposes, this index is best for individuals within the same specialty.^6^ The h-index encompasses measurements of quantifying in conjunction with quality. Using the Scopus database, each DC's current h-index as of January 2021 were recorded.

Statistical analysis in the form of Student *t*-tests was used to compare the means between two groups, and *P* < 0.05 was considered significant. All statistical analysis was performed using GraphPad Prism version 8.0 (GraphPad).

## Results

**Figure 1:**
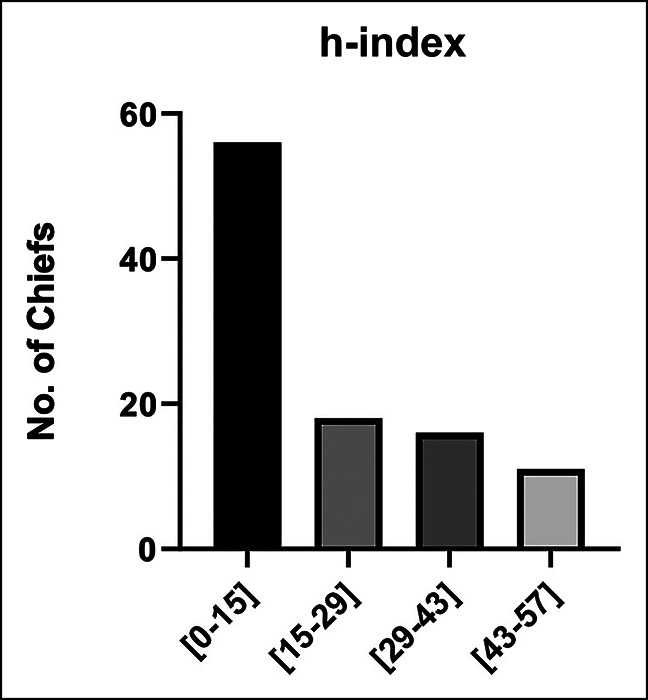
Bar chart showing h-indices of division chiefs.

**Figure 2:**
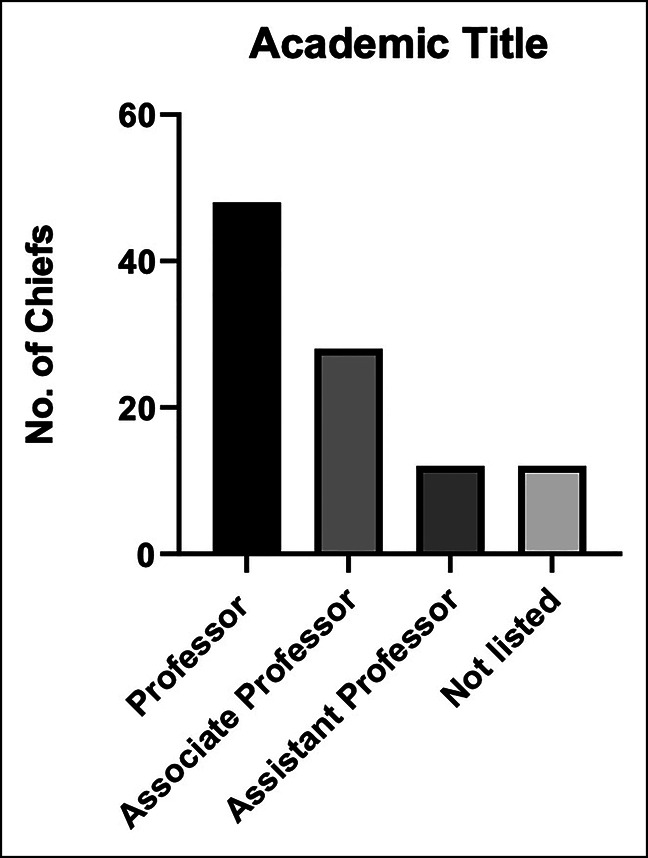
Bar chart showing academic title of division chief at their respective institution. “Not listed” indicates that information was not publicly available through the institution's website or that the position of division chief is not available at that institution.

Information on 100 DCs were obtained. Sixty-six of these programs offer sports medicine fellowship training at their institutions. These DCs consisted of 96% male and 4% female faculty. The racial/ethnic demographics of the DCs were mostly White (86%), followed by Asian (11%), African American (1%), Hispanic/Latino (1%), and mixed ethnicity (1%). The average number of faculty in each department was 8.5. The mean number of years since sports medicine fellowship completion was 19.6. The mean h-index was 21.2 (Table1). The DCs on average had a greater h-index among their subspecialty cohorts, 21.2 vs. 15.0 (*P* < 0.001). Most of the DC (56%) had an h-index between 0 and 15. Eighteen percent had an h-index between 15 and 29, 16% had an h-index between 29 and 43, and 11% had an h-index between 43 to 57 (Figure 1). Many (48%) hold an academic rank of professor, 28% an associate professor, and 12% an assistant professor, and 12% DCs did not have an academic rank listed (Figure 2).

**Table 1:** Demographics and Training

Demographics	
Men	96
Women	4
Mean h-index	21.2
Mean years since fellowship graduation	19.6
Mean number of faculty in division	8.5
Race/ethnicity (%)	
White	86
African American	1
Asian	11
Hispanic/Latino	1
Mixed	1

Forty-eight of the DCs held additional leadership positions within the orthopaedics department, within the hospital, or in their divisions. Twenty-one percent constituted of chairperson, 10.4% vice-chairperson, 12.5% director or associate director, 12.5% residency program directors, and 43% of fellowship director. Four of the DCs (4%) held additional graduate degrees (2 PhD, 1 JD, and 1 MS) (Table 2).

**Table 2:** Leadership and Graduate Degrees

Overall leadership	
Total sports medicine division chief	100
Programs offering fellowship	66
Concurrent leadership	
Chairperson	10
Vice-chairperson	5
Director or associate director of sports	6
Residency program director	6
Fellowship director	21
Additional degrees	
PhD	2
JD	1
Masters	1

**Figure 3:**
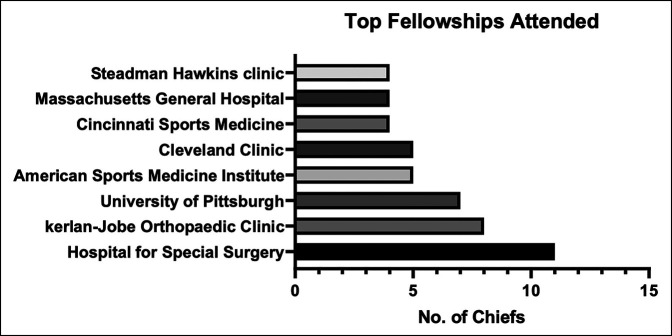
Bar chart showing institutions with at least 4 or more graduates who hold the position of sports medicine division chiefs.

Eight orthopaedic sports medicine fellowship programs were identified as having trained 4 or more orthopaedic sports medicine DC currently represented. These included Hospital for Special Surgery (n = 11), Kerlan Jobe Orthopaedics Clinic (n = 8), University of Pittsburg (n = 7), American Sports Medicine Institute (n = 5), and Cleveland Clinic (n = 5). Cincinnati Sports Medicine, Massachusetts General Hospital, and Steadman Hawkins Clinic all had 4 fellows currently serving as DCs (Figure 3).

**Figure 4:**
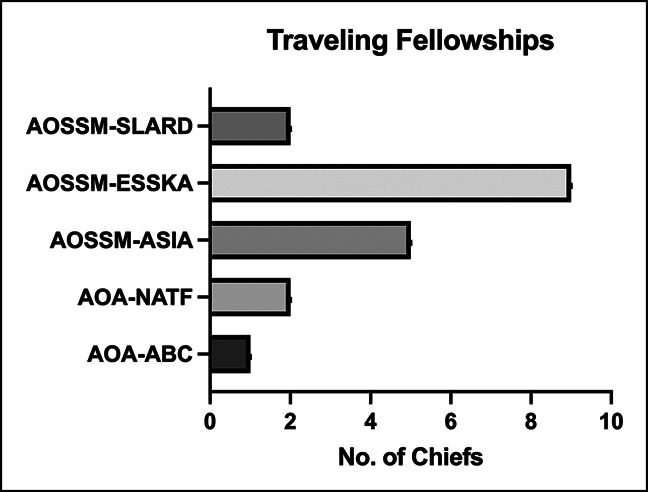
Bar chart showing traveling fellowships attended by division chiefs. AOA-ABC = American Orthopaedic Association, American British Canada Traveling Fellowship, AOA-NATF = American Orthopaedic Association, North American Traveling Fellowship, AOSSM-ASIA = American Orthopaedic Society for Sports Medicine, ASIA, AOSSM-ESSKA = American Orthopaedic Society for Sports Medicine, European Society for Sports Traumatology, Knee Surgery and Arthroscopy, AOSSM-SLARD = American Orthopaedic Society for Sports Medicine, Latin American Society of Knee Arthroscopy and Sports Medicine.

Nineteen DCs attended a traveling fellowship during their careers (Figure 4): 9 completed AOSSM-ESSKA, 5 AOSSM-Asia, 2 AOSSM-SLAF, 2 AOA-NATF, and 1 AOA-ABC. DCs who completed a traveling fellowship were more likely to have higher h-indices (*P* < 0.001) and a higher number of publications (*P* < 0.001).

Among the DCs, 10 have been highlighted as being the most scholarly productive (Table 3). On average, they had a Scopus h-index of 48.1 ± 4.4 and 244.4 ± 75.4 average publications. The topmost productive DC had a Scopus h-index of 55, while the 10th most productive had a Scopus h-index of 44.

**Table 3:** Top 10 Most Productive Chiefs

Institution	Division Chief	h-index	No. of Publications
University of Michigan	Asheesh Bedi, MD	55	367
Vanderbilt University	Rick Wright, MD	55	254
University of Connecticut	Robert A. Arciero, MD	52	207
Columbia University	Christopher S. Ahmad MD	48	302
University of Southern California	James Tibone, MD	48	142
Stanford University	Marc R. Safran, MD	45	233
University of Pittsburgh	Volker Musahl, MD	45	295
University of Utah	Robert Burks, MD	45	138
Rush University	Nikhil N. Verma, MD	44	311
Ohio State University	Christopher Kaeding, MD	44	195

## Discussion

Currently, studies evaluating the academic and leadership characteristics of orthopaedic surgery DCs, including the leaders of sports medicine, are scarce. This is the first study investigating the academic and leadership characteristics of sports medicine DCs. The results of this study also highlight the lack of diversity among the leaders in sports medicine, the demographic characteristics showed that most were men (96%) and White (86%). The DCs were on average 19.6 years past fellowship completion, having high academic rankings (most were professors) and more likely to be scholarly productive. Most of the DCs did not have an additional graduate degree (96%). Almost half (48%) of the DCs held concurrent leadership position within the department or within the greater institution.

Leadership positions within the division provide a unique opportunity to influence the trajectory of the division by creating unique clinical, research, and educational programs.^7^ Donnally et al.^8^ analyzed the demographic and academic trends among spine fellowship leaders and reported that the fellowship leaders were more likely to graduate from select residency and fellowship programs. It is unclear whether this result is due to the training that a fellow or resident received at these institutions or the institution's predilection to seek applicants who are more academic and likely to pursue leadership positions. Our study indicates that select fellowship programs in sports medicine are more likely to have graduates in leadership positions. Within the spine division, reports by Post et al.^9^ indicate that the mean age index increases significantly as a faculty rank increases and a positive correlation exists between the h-index and the practice duration. In our study of the sports medicine DCs identified, many had an academic rank of professor (48%) and, on average, had higher h-indices (18.4) compared with that of general surgeons and other surgical subspecialties. Similar to our results, other specialties have conducted studies on division leaders. Plastic surgery studies showed that the field consists of mostly men and the DCs often hold an academic rank of professor.^10^

In this study, 19 of the DCs attended a traveling fellowship either with AOA or AOSSM. The opportunity to attend traveling fellowships provides valuable experiences to orthopaedic sports medicine surgeons. The AOA-NATF or AOA-ABC traveling fellowship offers one such opportunity for young and mid-career orthopaedic surgeons, respectively. It is designed to advance orthopaedic surgeon's advancing careers by promoting leadership development, network creation, and knowledge or scientific exchange among emerging leaders in orthopaedic surgery.^11^ The experience to see how leadership in orthopaedic surgery is led in different settings allows fellows to appreciate the different ways of balancing the intricacies of academic and clinical life and lessons that can be applied to their home institutions when they return.^12^ Similarly, the AOSSM traveling fellowship is an intercontinental exchange program, providing scientific exchange opportunities between orthopaedic sports medicine surgeons in North America and the Asia-Pacific. The participation in traveling fellowship fosters strong foundation in leadership and networking, setting the stage for leadership positions down the road in the respective person's academic career.

The demographic composition of orthopaedic surgery is lagging behind the steady increase in diversity across the United States. Data from the US Census Bureau, the Accreditation Council for Graduate Medical Education, and the American Academy of Orthopaedic Surgeon (AAOS) reveal that orthopaedic surgery is the least diverse of all surgical subspecialties.^13^ A study on the orthopaedic chairpersons and program directors found that 98% of chairpersons were men versus 88.8% of program directors and that chairpersons were more likely to have academic ranks of professorship and were more scholarly productive compared with the program directors.^14^ Sex disparities in grant funding, leadership positions, and publication impact have been reported among vascular surgeons.^15^ In academic cardiology, a lower percentage of women holding the DCs (5%) and fellowship directors (14%) have also been reported.^16^ Sex disparities in orthopaedic exist, where women are underrepresented in number, rank, and academic productivity.^17^ Our result showed that DCs in orthopaedic sports medicine consists of only 4% women and 14% minorities (1% Hispanic/Latino, 1% African American, 1% mixed, and 11% Asians). Improving the overall diversity of the profession will bring many positive changes, such as promoting innovation through the diversity of ideas and backgrounds. There are many avenues to achieve this aim; recruiting qualified diverse faculty for leadership positions is one way. This can affect positively in attracting more minority medical students to pursue careers in orthopaedics through pipeline programs, mentorship, and active recruiting. The minority leaders can provide guidance, mentorship, and community outreach. A diverse residency pool and increased diversity within the field will allow for diverse and talented leaders. Our study showed select fellowship programs that continue to graduate more DC than others. Another avenue to increase diversity in the sports medicine leadership is for these fellowship programs to make a conscious effort to increase diversity among their recruits for their respective sports medicine fellowship.

This study has several limitations. First, we could not locate every DC associated with every orthopaedic residency program at academic institutions because of the lack of this information on their respective publicly accessible websites. Second, this is a cross-sectional study, and the data gathered and represented portrays information available on the sites at the time of data acquisition. The authors only surveyed the current status of the many parameters that represent the DCs, and these parameters and qualification are on a continual change and not necessarily representative of the qualification of the DCs at the time of attaining the leadership position. Third, discussion is ongoing in health research about how to best report race/ethnicity.^18^ In this study, the authors identified race/ethnicity using the country of origin of the last name of the DC. This limitation may lead to the potential of the reported races in the study to be different from self-identified race/ethnicity by individual DCs. However, we attempted to minimize this limitation by using each DC profile picture to further help confirm their ethnicity. In addition, our results do not differ significantly from that reported by AAOS's demographic of all orthopaedic surgeons, where, in 2018, 84% of all orthopaedic surgeons self-identified as Caucasian, 6.7% as Asian, 2.2% as Hispanic/Latino, and 1.9% as African American.^19^ A continuous effort is needed to increase diversity in orthopaedic and specifically in orthopaedic sports medicine leadership.

In conclusion, this is the first article to identify several characteristics of academic orthopaedic sports medicine DCs. The Orthopaedic Sports Medicine DCs are predominately men, with strong academic productivity (h-index), academic rank of professor, and more likely to have a concurrent leadership position in the department or the institution they practice. An apparent lack of diversity exists in the leadership of academic orthopaedic sports medicine surgery. Based on our findings, these leaders of the field have graduated from a select number of fellowship institutions. Increased diversity awareness and recruitment by these fellowship institutions and orthopaedic residency programs are ways to improve diversity of sports medicine leaders in the future.
